# Phenolic Profiling and Bioactivity Evaluation of *Stachys aleurites* Extracts: LC–ESI–MS/MS Characterization, Antioxidant Capacity, and Enzyme Inhibitory Potential

**DOI:** 10.1002/cbdv.71679

**Published:** 2026-09-04

**Authors:** Fatma Ozlem Kargin Solmaz, Cengiz Sarikurkcu, Bektaş Tepe

**Affiliations:** ^1^ Faculty of Pharmacy Afyonkarahisar Health Sciences University Afyonkarahisar Türkiye; ^2^ Faculty of Science, Department of Molecular Biology and Genetics Kilis 7 Aralik University Kilis Türkiye

**Keywords:** antioxidant activity, enzyme inhibition, LC–ESI–MS/MS profiling, phenolic compounds, *Stachys aleurites*

## Abstract

Natural products rich in phenolic compounds are increasingly investigated as multifunctional agents against oxidative stress and enzyme‐related disorders. This study characterized the phytochemical composition and evaluated the antioxidant and enzyme inhibitory activities of methanol and aqueous extracts of *Stachys aleurites*. Total phenolic and flavonoid contents were determined spectrophotometrically, while selected phenolics were quantified by LC‐ESI‐MS/MS. Antioxidant and enzyme inhibitory properties were assessed using complementary in vitro assays.

The methanol extract contained higher levels of total phenolics (48.86 mg GAE/g) and flavonoids (65.36 mg RE/g) than the aqueous extract. Verbascoside and chlorogenic acid were the predominant compounds in the methanol extract, whereas *p*‐coumaric acid was relatively more abundant in the aqueous extract. The methanol extract exhibited stronger antioxidant activity in most assays and showed greater inhibition of AChE, tyrosinase, α‐amylase, and α‐glucosidase. In contrast, the aqueous extract displayed slightly higher metal‐chelating activity and stronger BChE inhibition. These findings demonstrate solvent‐dependent variations in phytochemical composition and bioactivity, highlighting the importance of extraction solvent selection and supporting further studies on the bioactive constituents of *S. aleurites*.

## Graphical abstract

Solvent polarity shapes the phytochemical and bioactivity profile of *Stachys aleurites*. Methanol extraction enriches phenolics, especially verbascoside and chlorogenic acid, and enhances antioxidant and enzyme inhibitory activities, whereas the aqueous extract shows selective metal‐chelating and BChE inhibitory potential.

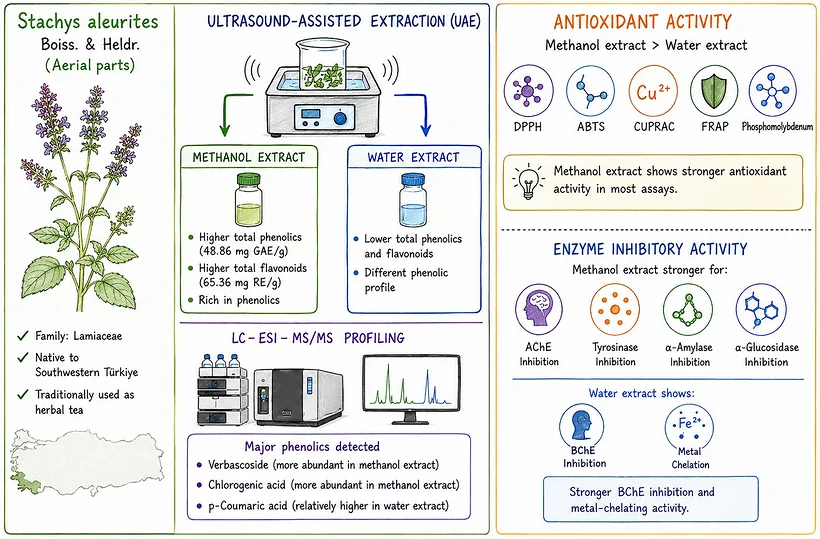

## 1 Introduction

Plants belonging to the Lamiaceae family are widely recognized as prolific producers of secondary metabolites with significant ecological and pharmacological functions. Members of this family are particularly rich in phenolic acids, flavonoids, and other bioactive compounds that contribute to diverse biological properties. Numerous phytochemical surveys have shown that Lamiaceae species frequently exhibit strong antioxidant capacity due to the abundance of phenolic constituents capable of scavenging free radicals and reducing oxidized intermediates [1]. In addition, phenolic compounds occurring in this family have been associated with a variety of biological effects, including anti‐inflammatory, antimicrobial, neuroprotective, and enzyme‐inhibitory activities [2, 3]. Such biochemical diversity has made Lamiaceae plants an important focus of phytochemical and pharmacological research.

Within this family, the genus *Stachys* represents one of the most species‐rich and chemically diverse groups. The genus comprises approximately 300 species distributed mainly across temperate regions of Europe, Asia, and the Mediterranean basin [4]. Phytochemical investigations of different *Stachys* species have revealed the presence of structurally diverse secondary metabolites, including flavonoids, phenolic acids, phenylethanoid glycosides, iridoids, diterpenoids, and triterpenoids [5]. Among these constituents, phenylethanoid glycosides such as verbascoside and related compounds are frequently reported as characteristic metabolites of the genus [4]. These compounds are often implicated in the pronounced antioxidant and enzyme‐modulating activities reported for several *Stachys* taxa.

Beyond their chemical diversity, many *Stachys* species have attracted attention because of their ethnomedicinal relevance. Several taxa are traditionally consumed as herbal infusions or used in folk medicine for the management of gastrointestinal disorders, inflammatory conditions, infections, and metabolic ailments [6]. Ethnobotanical surveys conducted in Türkiye indicate that the aerial parts of *Stachys* species are commonly harvested and prepared as herbal teas, highlighting the practical and cultural importance of these plant organs [6]. Such traditional practices also support the scientific exploration of aerial‐part extracts in phytochemical and biological screening studies.

Despite the increasing number of phytochemical investigations within the genus, knowledge regarding several individual species remains limited. *Stachys aleurites* Boiss. & Heldr., a species native to southwestern Türkiye, represents one such poorly characterized taxon. Earlier research on this plant has mainly focused on volatile constituents of the essential oil obtained from aerial parts, where compounds such as β‐caryophyllene, germacrene D, and bicyclogermacrene were reported as dominant components [7]. Additional work addressing Turkish *Stachys* taxa included determination of volatile profiles and total phenolic content in selected species, but detailed phytochemical profiling of *S. aleurites* remained very limited [8]. Furthermore, antimicrobial activity of extracts obtained from the aerial parts of the species has been evaluated against several microorganisms, indicating potential biological relevance [9]. Nevertheless, a comprehensive investigation integrating phenolic profiling with antioxidant and enzyme‐inhibitory activity assessment for this species has not been adequately documented in the literature.

Considering the phytochemical richness of the genus and the limited information available for *S. aleurites*, further investigation of this species appears warranted. In the present study, methanolic and aqueous extracts prepared from the aerial parts of *S. aleurites* were examined in order to obtain a broader understanding of its chemical composition and biological potential. Total phenolic and total flavonoid contents were determined spectrophotometrically, while selected phenolic compounds were quantified using LC‐ESI‐MS/MS analysis. In addition, antioxidant properties were evaluated through multiple complementary assays, including phosphomolybdenum, ferrous ion chelating, DPPH, ABTS, CUPRAC, and FRAP tests. The enzyme‐inhibitory potential of the extracts was also assessed against acetylcholinesterase (AChE), butyrylcholinesterase (BChE), α‐amylase, α‐glucosidase, and tyrosinase. By integrating phytochemical profiling with a broad panel of biological assays, the present work aims to provide a more comprehensive characterization of *S. aleurites* and to contribute baseline data for future phytochemical, pharmacological, and chemotaxonomic studies within the genus.

## 2 Results and Discussion

### 2.1 Chemical Composition

**FIGURE 1:**
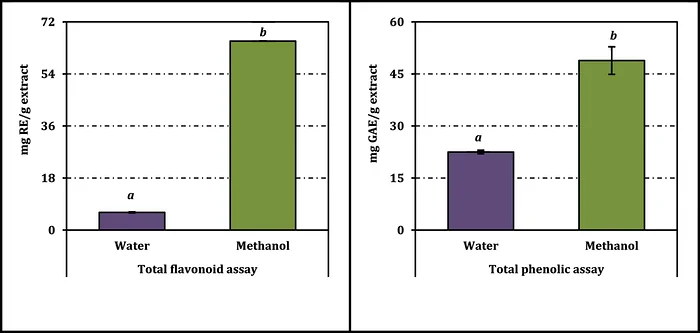
Total flavonoid and phenolic contents of *S. aleurites* extracts. The mean values followed by the same superscripts (a and b) do not differ, according to the Student's *t*‐test at the 5% significance level. REs (rutin equivalents) and GAEs (gallic acid equivalents).

The phytochemical profiling of *S. aleurites* extracts revealed a marked solvent‐dependent variation in both global phenolic metrics and individual phenolic constituents. As shown in Figure 1, the methanol extract exhibited significantly higher total phenolic and total flavonoid contents than the water extract, reaching 48.86 mg GAE/g extract and 65.36 mg RE/g extract, respectively, whereas the corresponding values for the water extract were 22.48 mg GAE/g extract and 6.11 mg RE/g extract. These results indicate that methanol was substantially more effective than water for recovering the phenolic and especially flavonoid fraction of the plant material.

The targeted LC‐ESI‐MS/MS data further supported this distinction and demonstrated that the methanol extract was characterized by a much richer accumulation of several major phenolics (Table 1). Verbascoside was the predominant compound in the methanol extract (7148 µg/g extract) and was not detected in the water extract. Likewise, chlorogenic acid was abundant in the methanol extract (5437 µg/g extract) but occurred only at a trace level in the water extract (18.4 µg/g extract). Methanol also extracted notably higher amounts of luteolin 7‐glucoside, protocatechuic acid, 2,5‐dihydroxybenzoic acid, caffeic acid, hyperoside, luteolin, and kaempferol. Among these, the differences for chlorogenic acid and luteolin‐derived constituents were particularly pronounced, highlighting the affinity of methanol for flavonoid glycosides and related phenolic metabolites.

**TABLE 1:** Concentration (µg/g extract) of selected phenolic compounds in *S. aleurites* extracts.

Compounds	Methanol	Water
Verbascoside	7148 ± 4	nd
Chlorogenic acid	5437 ± 18*^a^*	18.4 ± 0.3*^b^*
Luteolin 7‐glucoside	177 ± 6*^a^*	24.1 ± 0.1*^b^*
Protocatechuic acid	81.5 ± 1.7*^a^*	12.2 ± 0.2*^b^*
2,5‐Dihydroxybenzoic acid	81.9 ± 2.2*^a^*	12.2 ± 0.1*^b^*
Caffeic acid	69.1 ± 0.2*^a^*	0.79 ± 0.03*^b^*
Hyperoside	64.7 ± 0.1*^a^*	1.55 ± 0.04*^b^*
Syringic acid	62.1 ± 5.0*^a^*	59.0 ± 0.3*^a^*
Luteolin	44.4 ± 0.1*^a^*	7.38 ± 0.12*^b^*
Kaempferol	40.0 ± 0.1*^a^*	15.2 ± 0.1*^b^*
*p*‐Coumaric acid	26.0 ± 0.4*^b^*	200 ± 1*^a^*
Apigenin	20.9 ± 0.1*^a^*	14.2 ± 0.2
4‐Hydroxybenzoic acid	17.6 ± 0.3*^b^*	41.0 ± 0.2*^a^*
3‐Hydroxybenzoic acid	17.4 ± 0.4*^b^*	39.5 ± 0.4*^a^*
Ferulic acid	15.5 ± 0.3*^b^*	24.5 ± 1.0*^a^*
Vanillin	12.4 ± 0.1*^a^*	9.17 ± 0.11*^b^*
Rosmarinic acid	5.71 ± 0.16*^b^*	24.5 ± 0.2*^a^*
Hesperidin	4.80 ± 0.07	nd
Apigenin 7‐glucoside	4.63 ± 0.02*^b^*	8.63 ± 0.05*^a^*
Quercetin	2.12 ± 0.08	nd
(‐)‐Epicatechin	nd	nd
(+)‐Catechin	nd	nd
2‐Hydroxycinnamic acid	nd	nd
3,4‐Dihydroxyphenylacetic acid	nd	nd
Eriodictyol	nd	nd
Gallic acid	nd	nd
Pinoresinol	nd	nd
Pyrocatechol	nd	nd
Sinapic acid	nd	nd
Taxifolin	nd	nd

The mean values followed by the same superscripts (a and b) within a row do not differ, according to the Student's t‐test at 5% significance level. nd: Not detected.

In contrast, the water extract contained higher levels of a more limited set of phenolics, most notably *p*‐coumaric acid, which reached 200 µg/g extract compared with 26.0 µg/g extract in the methanol extract (Table 1). Water also yielded significantly higher concentrations of 4‐hydroxybenzoic acid, 3‐hydroxybenzoic acid, ferulic acid, rosmarinic acid, and apigenin 7‐glucoside. Syringic acid was quantified at comparable levels in both extracts, with no statistically significant difference between them. Some compounds, including hesperidin and quercetin, were detected only in the methanol extract, whereas several analytes such as (‐)‐epicatechin, (+)‐catechin, gallic acid, taxifolin, sinapic acid, eriodictyol, and pinoresinol were not detected in either extract.

The phytochemical data indicate that methanol provided broader and more efficient recovery of the selected phenolic constituents of *S. aleurites*, with particularly strong enrichment in verbascoside and chlorogenic acid, while the water extract was relatively enriched in specific hydroxybenzoic and hydroxycinnamic acid derivatives (Figure S1; Table 1). This compositional divergence suggests that solvent polarity critically shaped the extract profiles and is likely to influence their subsequent biological properties.

An important observation is that extraction yield and phenolic recovery did not follow the same trend. Although the aqueous extract provided a higher overall extraction yield (20.13%) than the methanol extract (15.80%), it contained substantially lower total phenolic and flavonoid contents. This discrepancy indicates that the higher yield of the aqueous extract was likely associated with the co‐extraction of highly polar non‐phenolic constituents, including sugars, amino acids, organic acids, and other water‐soluble matrix components. Consequently, extraction yield alone should not be considered a direct indicator of phenolic recovery or biological potential. Instead, the chemical composition of the recovered fraction appears to be a more relevant determinant of extract quality and subsequent bioactivity.

The present study provides the first comprehensive characterization of the non‐volatile phenolic profile of *S. aleurites*, thereby addressing a clear gap in the existing literature. Previous investigations on this species have been limited to its essential oil composition and seed oil constituents, including fatty acids and tocopherols [7, 8, 10]. These studies consistently highlighted sesquiterpene hydrocarbons such as β‐caryophyllene and bicyclogermacrene as dominant volatile constituents, alongside linoleic acid‐rich seed oils. However, no prior research has explored the phenolic metabolite spectrum of this species. Accordingly, the current findings establish a novel phytochemical baseline for *S. aleurites*, expanding its chemical characterization beyond lipophilic and volatile fractions to include polar bioactive constituents.

A key outcome of this study is the pronounced solvent‐dependent differentiation in phenolic composition, with methanol proving markedly more efficient in extracting a broader and more diverse set of phenolics, particularly flavonoid glycosides and phenylethanoid derivatives. This observation is consistent with the well‐documented affinity of mid‐polar organic solvents for compounds such as verbascoside and flavonoid conjugates, which are poorly recovered in aqueous systems due to differences in solubility and matrix interactions. Comparable solvent‐dependent patterns have also been reported in other *Stachys* species, where methanolic extracts were shown to concentrate major phenolics such as chlorogenic acid, verbascoside, and flavonoid derivatives more effectively than water extracts [11, 12, 13].

The dominance of verbascoside in the methanol extract is particularly noteworthy, as phenylethanoid glycosides of this type are recurrent constituents within the genus *Stachys* and more broadly within Lamiaceae. Recent comparative and review‐based evidence indicates that verbascoside and related phenylethanoid glycosides are widely distributed across *Stachys* species, while chlorogenic acid and flavonoids also represent major non‐volatile constituents in several species [5, 14]. This broader phytochemical context supports the view that *S. aleurites* shares core phenylpropanoid biosynthetic features with other members of the genus. The concurrent detection of chlorogenic acid and luteolin‐derived constituents in the present study is also in line with reports from other *Stachys* species, including *S. lavandulifolia* and *S. rizeensis*, where hydroxycinnamic acid derivatives (such as chlorogenic acid) and flavonoids (including luteolin‐type compounds) were likewise found to be prominent components [13, 15].

Comparative analysis with other *Stachys* species indicates that, although the qualitative phenolic framework is broadly shared across the genus, the relative abundance of individual constituents may vary substantially according to species, plant organ, and extraction conditions. Studies on *S. lavandulifolia* and *S. cretica* subsp. *kutahyensis* have shown that hydroxycinnamic acid derivatives and flavonoid‐type constituents are prominent components, while their relative distribution differs depending on the taxon and solvent system employed [11, 15]. Consistent with this pattern, the relatively higher enrichment of certain hydroxycinnamic acids and simple phenolics in the water extract suggests that more polar, lower‐molecular‐weight constituents were recovered more efficiently under aqueous extraction conditions.

The phytochemical profile obtained in this study not only complements the previously reported volatile and lipid fractions of *S. aleurites* but also positions this species within the broader chemotaxonomic framework of the genus *Stachys*. Overall, the marked influence of solvent polarity on extract composition underscores the critical role of extraction strategy in shaping phytochemical profiles and highlights the need to tailor solvent systems according to the targeted class of bioactive compounds.

### 2.2 Antioxidant Activity

**FIGURE 2:**
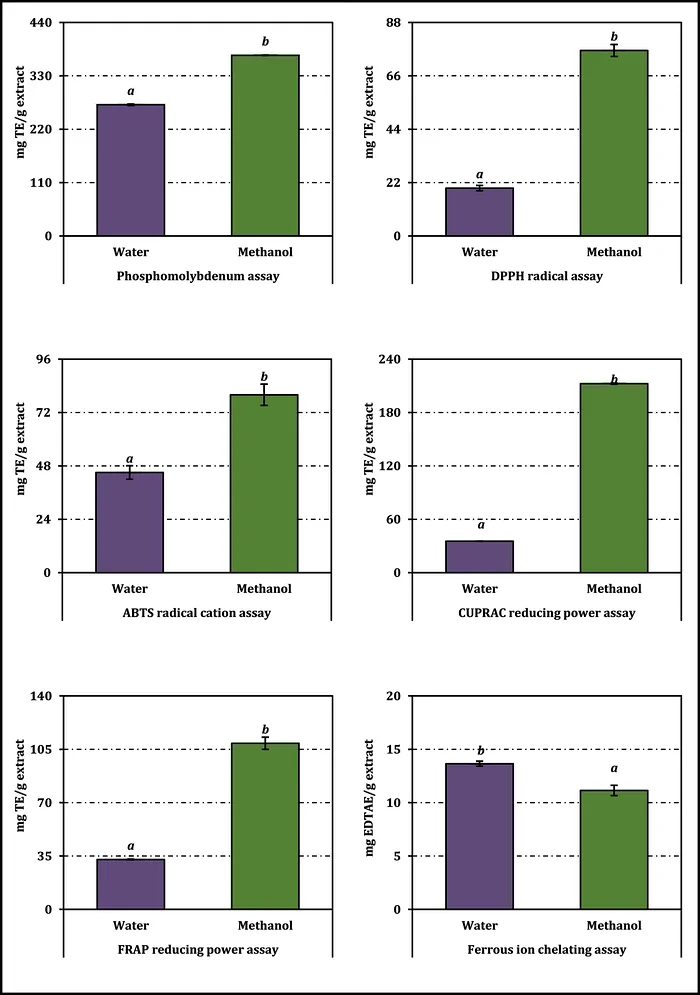
Antioxidant activity of *S. aleurites* extracts evaluated by DPPH (2,2‐diphenyl‐1‐picrylhydrazyl radical scavenging), ABTS (2,2′‐azinobis‐(3‐ethylbenzothiazoline‐6‐sulfonic acid) radical cation scavenging), CUPRAC (cupric reducing antioxidant capacity), FRAP (ferric reducing antioxidant power), phosphomolybdenum, and ferrous ion chelating assays. The mean values followed by the same superscripts (a and b) do not differ, according to the Student's *t*‐test at the 5% significance level. TEs (Trolox equivalents) and EDTAEs (ethylenediaminetetraacetic acid disodium salt equivalents), respectively.

The antioxidant potential of the *S. aleurites* extracts was evaluated using complementary radical scavenging, metal chelating, and reducing power assays. The quantitative outcomes expressed as IC_50_ or EC_50_ values are summarized in Table 2, while the same activities are additionally presented as positive control equivalents in Figure 2 to facilitate comparative interpretation.

**TABLE 2:** Antioxidant activities of *S. aleurites* extracts.

Assays	Methanol	Water	Trolox	EDTA
DPPH radical scavenging (IC_50_: mg/mL)	2.62 ± 0.08*^b^*	10.16 ± 0.56*^c^*	0.20 ± 0.03*^a^*	—
ABTS radical cation scavenging (IC_50_: mg/mL)	2.51 ± 0.15*^b^*	4.45 ± 0.30*^c^*	0.19 ± 0.03*^a^*	—
Ferrous ion chelating (IC_50_: mg/mL)	1.71 ± 0.07*^c^*	1.39 ± 0.02*^b^*	—	0.022 ± 0.003*^a^*
Phosphomolybdenum (EC_50_: mg/mL)	1.07 ± 0.01*^b^*	1.48 ± 0.01*^c^*	0.41 ± 0.02*^a^*	—
CUPRAC reducing power (EC_50_: mg/mL)	0.80 ± 0.02*^b^*	4.80 ± 0.02*^c^*	0.18 ± 0.02*^a^*	—
FRAP reducing power (EC_50_: mg/mL)	0.41 ± 0.02*^b^*	1.38 ± 0.01*^b^*	0.044 ± 0.003*^a^*	—

The mean values followed by the same superscripts (a, b and c) within the same row do not differ, according to the Tukey's honestly significant difference post hoc test at 5% significance level. EC_50_ (mg/mL), effective concentration at which the absorbance was 0.5 for reducing power and phosphomolybdenum assays. IC_50_ (mg/mL), inhibition concentration at which 50% of the DPPH and ABTS radicals were scavenged and the ferrous ion‐ferrozine complex were inhibited. EDTA, ethylenediaminetetraacetic acid (disodium salt). “‐”, not determined.

In the radical scavenging assays, the methanol extract displayed stronger activity than the water extract. For DPPH radical scavenging, the methanol extract yielded an IC_50_ value of 2.62 mg/mL, whereas the water extract required a substantially higher concentration (IC_50_ = 10.16 mg/mL) to achieve the same effect (Table 2). A similar trend was observed for ABTS radical cation scavenging, where the methanol extract (IC_50_ = 2.51 mg/mL) demonstrated nearly twofold greater activity than the water extract (IC_50_ = 4.45 mg/mL). In both assays, the reference antioxidant Trolox exhibited markedly lower IC_50_ values (0.20 and 0.19 mg/mL for DPPH and ABTS, respectively), indicating substantially higher radical scavenging efficiency than the plant extracts.

A different pattern emerged in the ferrous ion chelating assay. In this test, the water extract showed slightly stronger chelating capacity (IC_50_ = 1.39 mg/mL) compared with the methanol extract (IC_50_ = 1.71 mg/mL) (Table 2). Nevertheless, both extracts were considerably less active than the reference chelator EDTA, which displayed a very low IC_50_ value of 0.022 mg/mL.

The reducing power–based assays further highlighted the superior antioxidant capacity of the methanol extract. In the phosphomolybdenum assay, the methanol extract exhibited an EC_50_ value of 1.07 mg/mL, whereas the water extract showed lower activity (EC_50_ = 1.48 mg/mL). A more pronounced difference was observed in the CUPRAC assay, where the methanol extract (EC_50_ = 0.80 mg/mL) displayed approximately sixfold stronger reducing capacity than the water extract (EC_50_ = 4.80 mg/mL). In the FRAP assay, both extracts demonstrated comparable reducing power, with EC_50_ values of 0.41 mg/mL for the methanol extract and 1.38 mg/mL for the water extract (Table 2). As expected, Trolox consistently showed significantly stronger reducing activity in all assays.

**FIGURE 3:**
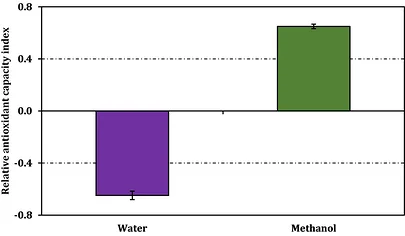
Relative antioxidant capacity index of *S. aleurites* extracts.

To provide an integrated assessment of antioxidant performance across the different assays, the results were further evaluated using the Relative Antioxidant Capacity Index (RACI). As shown in Figure 3, the methanol extract exhibited a positive RACI value of 0.65 ± 0.02, whereas the water extract showed a corresponding negative value of −0.65 ± 0.03. The higher RACI score of the methanol extract indicates its superior overall antioxidant performance when the outcomes of the individual antioxidant assays are considered collectively. This integrated evaluation is consistent with the individual assay results, in which the methanol extract generally displayed stronger radical scavenging and reducing capacities, despite the slightly greater ferrous ion chelating activity of the water extract.

The antioxidant profile of *S. aleurites* extracts indicates that the methanol extract generally possesses higher radical scavenging and reducing capacities, whereas the water extract exhibits slightly stronger metal chelating activity. For ease of comparison with standard antioxidants, the activities of the extracts are also expressed as Trolox or EDTA equivalents in Figure 2.

To the best of current knowledge, this study represents the first report describing the antioxidant potential of *S. aleurites*, thereby providing a novel contribution to the phytochemical and bioactivity profile of this species. The overall antioxidant behavior observed here reveals a clear solvent‐dependent pattern, with the methanol extract generally exhibiting superior radical scavenging and reducing capacities, whereas the water extract showed relatively better performance in metal chelation. This divergence likely reflects differences in the polarity‐driven extraction of phenolic subclasses with distinct redox mechanisms.

The stronger performance of the methanol extract in radical scavenging and reducing power assays can be directly associated with its higher total phenolic and flavonoid contents, as well as its richer profile of individual phenolic constituents. This observation is consistent with the widely accepted role of phenolic compounds as primary contributors to antioxidant activity through hydrogen atom transfer and single electron transfer mechanisms. A similar solvent‐dependent trend has been reported for *S. annua*, where the methanol extract demonstrated higher antioxidant activity than the aqueous extract across multiple assays, largely attributed to its enhanced phenolic content [16]. The agreement between these findings suggests that extraction efficiency of moderately polar phenolics is a key determinant of antioxidant performance within the genus.

Comparative insights from other *Stachys* species further support the relationship between phenolic composition and antioxidant capacity. For instance, species such as *S. germanica*, *S. palustris*, and *S. byzantina* have been identified as particularly active in DPPH and FRAP assays, with this activity generally associated with their higher total phenolic and flavonoid contents [17]. Likewise, decoctions of *S. byzantina* and *S. lavandulifolia* demonstrated strong radical scavenging activity and reducing power, again correlating with their phenolic richness [18]. In this context, the antioxidant profile of *S. aleurites* appears to align with the broader chemotaxonomic pattern observed in the genus, where phenolic abundance and composition govern bioactivity outcomes.

Interestingly, the relatively higher metal chelating activity observed in the water extract suggests the presence of more hydrophilic constituents with metal‐binding capacity, such as certain phenolic acids, sugars, or other polar metabolites. This behavior is not uncommon in plant extracts, as different antioxidant assays probe distinct mechanisms, and a lack of direct correlation between them has been frequently reported. Indeed, Benedec et al. [17] highlighted that radical scavenging and reducing power assays do not always correlate with metal chelation or lipid peroxidation inhibition within *Stachys* species, emphasizing the multifaceted nature of antioxidant activity.

When compared to other members of the genus, the antioxidant capacity of *S. aleurites* can be considered moderate rather than exceptional. For example, in *S. spectabilis*, both the aqueous extract and its derived silver nanoparticles exhibited notable radical scavenging activity, with the nanoparticle formulation showing enhanced performance due to improved electron transfer and stabilization mechanisms [19]. Similarly, hydroalcoholic extracts of *S. pilifera* have demonstrated measurable reducing power, although not always significantly different from control systems at certain concentrations [20]. These variations across species and extraction systems further underline that antioxidant potential in *Stachys* is highly dependent on both phytochemical composition and extraction strategy.

The findings indicate that *S. aleurites* possesses moderate antioxidant activity across different assay systems, likely associated with its phenolic constituents. The observed solvent‐dependent differences reinforce the importance of extraction conditions in maximizing bioactive compound recovery.

### 2.3 Enzyme Inhibition Activity

**FIGURE 4:**
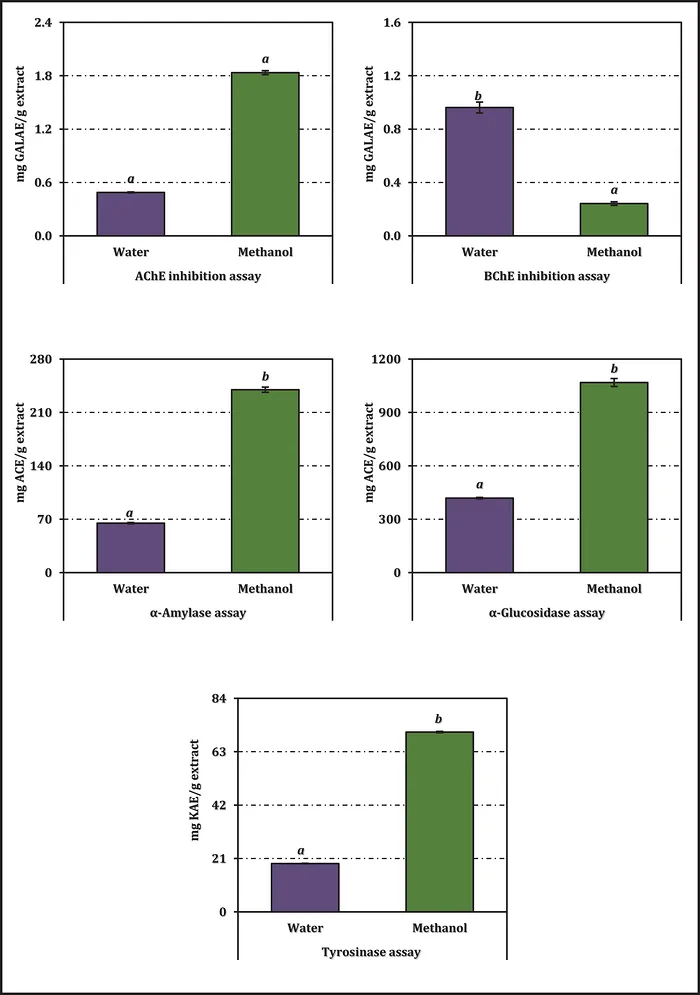
Enzyme inhibitory activity of *S. aleurites* extracts against acetylcholinesterase (AChE), butyrylcholinesterase (BChE), α‐amylase, α‐glucosidase, and tyrosinase. The mean values followed by the same superscripts (a and b) do not differ, according to the Student's *t*‐test at the 5% significance level. ACEs (acarbose equivalents), GALAEs (galanthamine equivalents), and KAEs (kojic acid equivalents), respectively.

The inhibitory effects of the methanol and water extracts of *S. aleurites* against selected enzymes related to neurodegenerative disorders, skin hyperpigmentation, and carbohydrate metabolism are summarized in Table 3. For complementary visualization, the activities expressed as positive control equivalents are also presented in Figure 4.

**TABLE 3:** Enzyme inhibition activity of *S. aleurites* extracts.

Samples	Methanol	Water	Galanthamine	Kojic acid	Acarbose
AChE inhibition (IC_50_: mg/mL)	1.53 ± 0.02*^b^*	5.76 ± 0.06*^c^*	0.0027 ± 0.0002*^a^*	—	—
BChE inhibition (IC_50_: mg/mL)	12.90 ± 0.72*^c^*	3.25 ± 0.14*^b^*	0.0032 ± 0.0003*^a^*	—	—
Tyrosinase inhibition (IC_50_: mg/mL)	1.06 ± 0.01*^b^*	3.95 ± 0.02*^c^*	—	0.076 ± 0.004*^a^*	—
α ‐Amylase inhibition (IC_50_: mg/mL)	3.85 ± 0.05*^b^*	14.25 ± 0.19*^c^*	—	—	0.96 ± 0.05*^a^*
α ‐Glucosidase inhibition (IC_50_: mg/mL)	1.17 ± 0.02*^a^*	2.99 ± 0.03*^b^*	—	—	1.24 ± 0.04*^a^*

Values indicated by the same superscripts (a–c) within the same row are not different from the honestly significant difference after Tukey's hoc test at 5% significance level. IC_50_ (mg/mL), inhibition concentration at which 50% of the enzymes were inhibited.

Regarding cholinesterase inhibition, the methanol extract exhibited stronger acetylcholinesterase (AChE) inhibition with an IC_50_ value of 1.53 mg/mL, whereas the water extract showed a markedly weaker effect (IC_50_ = 5.76 mg/mL) (Table 3). In contrast, butyrylcholinesterase (BChE) inhibition followed an opposite trend: the water extract demonstrated greater activity (IC_50_ = 3.25 mg/mL) compared with the methanol extract (IC_50_ = 12.90 mg/mL). As expected, the reference inhibitor galanthamine showed substantially stronger inhibition for both enzymes.

The extracts also displayed measurable inhibitory activity against tyrosinase. The methanol extract was more potent (IC_50_ = 1.06 mg/mL) than the water extract (IC_50_ = 3.95 mg/mL) (Table 3). Nevertheless, both extracts were considerably less active than the standard kojic acid.

In terms of carbohydrate‐hydrolyzing enzymes, the methanol extract again showed stronger α‐amylase inhibition (IC_50_ = 3.85 mg/mL) than the water extract (IC_50_ = 14.25 mg/mL), although both were weaker than the reference drug acarbose. For α‐glucosidase inhibition, however, the methanol extract (IC_50_ = 1.17 mg/mL) displayed inhibition approaching that of acarbose under the experimental conditions employed (IC_50_ = 1.24 mg/mL), while the water extract showed lower potency (IC_50_ = 2.99 mg/mL) (Table 3).

The results indicate that the methanol extract generally exerted stronger inhibitory effects against AChE, tyrosinase, α‐amylase, and α‐glucosidase, whereas the water extract was more effective against BChE. The corresponding activities expressed as acarbose, galanthamine, and kojic acid equivalents are provided in Figure 4 to facilitate comparative visualization of the enzyme inhibition profiles.

The present findings provide the first evidence on the enzyme inhibitory potential of *S. aleurites*, thereby expanding the bioactivity profile of this species beyond its previously reported phytochemical and antioxidant properties. Given the absence of prior data for this taxon, a meaningful interpretation of the results can be achieved through comparison with closely related *Stachys* species, particularly in the context of extraction solvent and phenolic composition.

A clear solvent‐dependent pattern was observed, with the methanol extract generally exhibiting stronger inhibitory effects against AChE, tyrosinase, α‐amylase, and α‐glucosidase. This trend is consistent with previous reports on *Stachys* species, where organic solvent extracts—especially methanol—tend to concentrate phenolic constituents with recognized enzyme inhibitory properties. For instance, methanol extracts of *S. cretica* subsp. *vacillans* displayed superior α‐amylase and tyrosinase inhibition compared to aqueous extracts, highlighting the role of solvent polarity in extracting bioactive compounds [21]. Similarly, in *S. chasmosericea*, methanol extracts showed consistently stronger inhibition across multiple enzymes than water extracts [22]. These observations support the notion that the higher phenolic and flavonoid content of the methanol extract in the present study likely underpins its enhanced inhibitory activity.

The relatively moderate cholinesterase inhibition observed for both extracts aligns with literature data indicating that *Stachys* species generally exhibit weaker anticholinesterase effects compared to their antidiabetic or anti‐tyrosinase activities. For example, both methanol and water extracts of *S. annua* demonstrated comparatively low inhibition of AChE and BChE despite showing strong activity against carbohydrate‐hydrolyzing enzymes [16]. A similar pattern was reported for *S. bombycina*, where both extracts exhibited limited cholinesterase inhibition, although the aqueous extract showed relatively higher selectivity toward BChE [23]. The latter observation is particularly noteworthy, as it parallels the present findings where the water extract of *S. aleurites* demonstrated comparatively stronger BChE inhibition. This may suggest that more polar constituents, potentially including certain glycosylated phenolics, contribute preferentially to BChE inhibition.

Regarding tyrosinase inhibition, the higher activity of the methanol extract is in agreement with previous studies on *Stachys* taxa. Methanol extracts of *S. germanica* subsp. *heldreichii* have been reported to exhibit notable tyrosinase inhibitory effects [12], and herbal infusions from *Stachys* species have shown strong anti‐tyrosinase potential expressed in kojic acid equivalents [18]. These findings collectively indicate that phenolic‐rich extracts from *Stachys* species may exhibit moderate tyrosinase inhibitory activity, likely due to the presence of compounds capable of interacting with copper ions at the enzyme's active site. However, their activity remains considerably lower than that of the reference inhibitor kojic acid.

In terms of antidiabetic potential, the observed inhibitory effects against α‐amylase and α‐glucosidase are also consistent with the general bioactivity profile of the genus. Some *Stachys* extracts have been reported to inhibit carbohydrate‐hydrolyzing enzymes more strongly than cholinesterases, although this pattern is not uniform across species [16, 23]. Notably, the methanol extract exhibited appreciable α‐glucosidase inhibition, approaching that of acarbose under the experimental conditions employed. However, since crude extracts contain chemically diverse constituents with different molecular weights and relative abundances, this result should not be interpreted as direct equivalence to the pure reference drug. This finding warrants further investigation through bioassay‐guided fractionation and mechanistic studies. This behavior is in line with findings from *S. annua*, where both aqueous and methanolic extracts exhibited potent α‐glucosidase inhibition [16]. Additionally, the differential selectivity between α‐amylase and α‐glucosidase inhibition observed across *Stachys* species [23] suggests that subtle variations in phytochemical composition can significantly influence enzyme targeting profiles.

The enzyme inhibitory activities of *S. aleurites* extracts appear to follow patterns commonly reported within the genus, particularly in terms of solvent‐dependent efficacy and stronger inhibition of carbohydrate‐hydrolyzing and tyrosinase enzymes relative to cholinesterases. However, the contribution of individual compounds to the observed enzyme inhibitory activities remains unknown. Therefore, the present findings should not be interpreted as evidence that any specific constituent, including the major compounds identified in the extracts, is directly responsible for the observed effects. Further studies involving bioactivity‐guided fractionation and testing of isolated compounds are required to clarify compound–activity relationships.

An additional aspect that enhances the significance of the present study is the limited phytochemical and biological information previously available for *S. aleurites*. While numerous *Stachys* species have been investigated with respect to phenolic composition, antioxidant properties, and enzyme inhibitory activities, comparable information for *S. aleurites* has remained largely unavailable. Therefore, beyond documenting the biological activities of the species, the present work establishes a reference dataset for future comparative studies within the genus. The predominance of verbascoside together with chlorogenic acid and luteolin‐derived constituents further supports the chemotaxonomic affinity of *S. aleurites* with other phenylpropanoid‐rich *Stachys* taxa, while also expanding current knowledge of species‐specific phytochemical variation within the genus.

## 3 Conclusions

The present study provides the first comprehensive characterization of the non‐volatile phenolic composition, antioxidant properties, and enzyme inhibitory activities of *S. aleurites*, revealing a clear solvent‐dependent pattern. The methanol extract contained higher levels of total phenolics and flavonoids and generally exhibited stronger radical scavenging, reducing power, and inhibition of AChE, tyrosinase, α‐amylase, and α‐glucosidase, whereas the water extract showed slightly stronger metal chelating activity and BChE inhibition. Verbascoside was the predominant constituent of the methanol extract, while the identified phenolic profile provides additional chemotaxonomic information for the genus *Stachys*. Overall, these findings expand the limited phytochemical and bioactivity data available for *S. aleurites* and provide a basis for further investigation of its bioactive constituents. However, as the biological activities were evaluated only at the extract level using in vitro assays, further studies involving isolation of active compounds, mechanistic evaluation, and appropriate safety and biological models are required before their potential applications can be established.

## 4 Materials and Methods

### 4.1 Plant Material

The aerial parts of *S. aleurites* were harvested during the flowering stage on April 21, 2023, from rocky slopes near Topçam Beach in the Konyaaltı district of Antalya Province, Türkiye (36°48′41″ N, 30°35′08″ E) at an elevation of approximately 30 m above sea level. Botanical identification of the collected specimens was performed by Dr. Olcay Ceylan (Department of Biology, Division of Botany, Muğla Sıtkı Koçman University, Muğla, Türkiye) (Herbarium number: O.2387).

### 4.2 Solvent Extraction

Collected aerial plant materials were air‐dried under shade in a well‐ventilated environment until a stable weight was achieved. The dried samples were then cut into small fragments using a laboratory blender and subsequently pulverized into a fine powder. This powdered material served as the starting material for extraction.

Ultrasound‐assisted extraction (UAE) was conducted using methanol and water as extraction solvents while maintaining a constant plant material–to–solvent ratio of 1:20 (w/v). For each extraction process, 5 g of powdered plant material was treated with 100 mL of solvent in an ultrasonic bath operating at approximately 40 kHz and 260 W at about 30°C for 60 min.

Ultrasound‐assisted extraction (UAE) was conducted using methanol and water as extraction solvents while maintaining a constant plant material–to–solvent ratio of 1:20 (w/v). Methanol and water were selected to represent two commonly used extraction systems with different polarities and to enable comparison between a polar organic solvent and a traditional aqueous preparation. Methanol is widely employed for efficient recovery of phenolic compounds, whereas water reflects the conventional mode of preparation frequently used for herbal infusions. Hydroalcoholic mixtures were not included in the present study because the primary objective was to compare the phytochemical and biological properties of extracts obtained using these two contrasting solvent systems. The UAE conditions (40 kHz, 60 min, approximately 30°C) were selected based on commonly applied parameters reported in previous phytochemical studies of *Stachys* species and related Lamiaceae taxa, providing efficient extraction while minimizing possible thermal degradation of phenolic constituents.

Following extraction, the mixtures were filtered through Whatman No. 1 filter paper. The aqueous extract was subsequently lyophilized, whereas the methanolic extract was concentrated under reduced pressure using a rotary evaporator. The resulting extracts were stored at 4°C until further analysis. Extraction yields for methanol and water extracts were calculated as 15.80% and 20.13%, respectively, expressed as percentage yield relative to the dry weight of plant material (w/w). The higher extraction yield obtained with water may be attributed to its greater ability to solubilize highly polar constituents such as sugars, organic acids, amino acids, and other water‐soluble matrix components. Consequently, although the aqueous extract exhibited a higher overall yield, this does not necessarily indicate a higher recovery of phenolic compounds, as reflected by its lower total phenolic and flavonoid contents compared with the methanol extract.

### 4.3 Determination of the Phenolic Compositions of the Extracts

Total phenolic content (TPC) was quantified using the Folin–Ciocalteu assay and reported as gallic acid equivalents (GAE), whereas total flavonoid content (TFC) was determined by the aluminium chloride colorimetric method and expressed as rutin equivalents (RE) [24, 25, 26].

To further characterize phenolic constituents, the extracts were analyzed using a previously validated LC–ESI–MS/MS method designed for simultaneous quantification of phenolic compounds [27]. Analyses were performed using an Agilent Technologies 1260 Infinity liquid chromatography system coupled with a 6420 Triple Quadrupole mass spectrometer.

Chromatographic separation was achieved on a Poroshell 120 EC‐C18 column (100 mm × 4.6 mm, 2.7 µm) employing gradient elution with solvent A (0.1% formic acid in water) and solvent B (methanol). The column temperature was maintained at 25°C, the flow rate was set to 0.4 mL min^−^^1^, and the injection volume was 2 µL.

Detection was performed using an electrospray ionization (ESI) source operating in multiple reaction monitoring (MRM) mode under both positive and negative ionization conditions. Identification and quantification of compounds were carried out by comparing retention times and characteristic ion transitions with those of authentic reference standards. The analytical validation parameters of the method are presented in Tables S1 and S2.

### 4.4 Antioxidant and Enzyme Inhibitory Assays

The antioxidant potential of the extracts was evaluated through a series of in vitro assays, including phosphomolybdenum total antioxidant capacity, DPPH and ABTS radical scavenging assays, ferrous ion chelating activity, cupric ion reducing antioxidant capacity (CUPRAC), and ferric reducing antioxidant power (FRAP), following previously established protocols [28, 29, 30, 31, 32, 33].

In the phosphomolybdenum assay, samples were reacted with a reagent mixture containing sulfuric acid, sodium phosphate, and ammonium molybdate, incubated at 95°C for 90 min, and the absorbance was recorded at 695 nm. DPPH radical scavenging activity was assessed by monitoring the decrease in absorbance of a methanolic DPPH solution at 517 nm after incubation with the extract under dark conditions.

The ABTS radical scavenging assay utilized the ABTS•^+^ radical cation generated by reacting ABTS with potassium persulfate, and absorbance was measured at 734 nm. Metal chelating activity was determined through the ferrozine–Fe^2^^+^ complex formation method at 562 nm. Reducing capacity was evaluated using CUPRAC and FRAP assays, with absorbance readings at 450 nm and 593 nm, respectively.

Enzyme inhibitory activities were investigated against α‐amylase, α‐glucosidase, tyrosinase, and cholinesterases (acetylcholinesterase and butyrylcholinesterase) using established methodologies [28, 32, 34]. α‐Amylase inhibition was determined using the Caraway–Somogyi iodine–potassium iodide method, whereas α‐glucosidase inhibition was measured using *p*‐nitrophenyl‐α‐D‐glucopyranoside (pNPG) as substrate. Tyrosinase inhibition was evaluated in a dopachrome‐based assay employing L‐DOPA as the substrate. Cholinesterase inhibitory activity was determined using Ellman's colorimetric assay with acetylthiocholine iodide or butyrylthiocholine chloride as substrates.

For radical scavenging, metal chelating, and enzyme inhibition assays, IC_50_ values were calculated as the concentration required to inhibit 50% of activity. For reducing power assays and the phosphomolybdenum test, EC_50_ values corresponded to the concentration producing an absorbance of 0.500. Biological activities were expressed as mg standard equivalents per g extract and compared with the activities of reference compounds used as positive controls. Detailed experimental procedures are provided in the Supporting Information.

### 4.5 Statistical Analysis

All experiments were conducted using three independent biological replicates (*n* = 3). For each solvent, the extraction procedure was performed independently three times, and the resulting extracts were analyzed separately. Therefore, the reported replicates represent independent biological replicates rather than analytical (technical) replicates. Results are presented as mean ± standard deviation (SD).

Statistical analyses were performed using IBM SPSS Statistics for Windows, Version 22.0 (IBM Corp., Armonk, NY, USA). Differences between two groups were assessed using Student's *t*‐test, whereas comparisons among multiple groups were analyzed using one‐way analysis of variance (ANOVA) followed by Tukey's post hoc test. Statistical significance was accepted at *p* < 0.05.

## Author Contributions

**Cengiz Sarikurkcu**: conceptualization, writing – original draft, writing –review and editing. **Bektaş Tepe**: methodology, writing –review and editing. **Fatma Ozlem Kargin Solmaz**: material preparation, data collection and analysis, writing – review and editing. The completed manuscript underwent comprehensive review by all authors, resulting in approval for publication. All authors have read and agreed to the published version of the manuscript.

## Funding

This research received financial support from the Scientific Research Projects Coordination Unit of Afyonkarahisar Health Sciences University under project number 25.GENEL.012.

## Ethics Statement

The authors have nothing to report.

## Conflicts of Interest

The authors declare no conflicts of interest.

## Use of Artificial Intelligence

Artificial intelligence (AI) tools, specifically OpenAI's ChatGPT (Generative Pre‐trained Transformer), were used to assist with language editing, conceptual clarity, manuscript presentation, and the preparation of the graphical abstract. AI assistance in the graphical abstract was limited to its visual design and presentation based exclusively on scientific content and results provided and verified by the authors. No AI tool was used for data generation, data analysis, interpretation of results, or scientific decision‐making. All experimental work, laboratory analyses, data generation, and scientific interpretation were carried out entirely by the authors. All AI‐assisted textual and visual content was carefully reviewed, verified, and approved by the authors to ensure scientific accuracy, transparency, and research integrity.

## Supporting information




**Supporting File 1**: cbdv71679‐sup‐0001‐SuppMat.docx

## Data Availability

Data will be made available on request.
